# Capsaicin as a Phytochemical Adjuvant Enhancing Cisplatin Efficacy in Nsclc: In Vitro and In Vivo Evidence

**DOI:** 10.3390/ph19060884

**Published:** 2026-06-01

**Authors:** Onur Onguncan, Nevruz Alis, Tulay Mutlu, Abdullah Yalcin, Oner Sonmez, Buse Birinci, Aybike Sarioglu-Bozkurt, Elif Bayram, Sabire Guler

**Affiliations:** 1 Department of Histology & Embryology, Faculty of Veterinary Medicine, Bursa Uludag University, Bursa 16059, Türkiye; onuronguncan@uludag.edu.tr; 2 Department of Histology & Embryology, Health Sciences Institute, Bursa Uludag University, Bursa 16059, Türkiye; nwrzalis@gmail.com (N.A.); tullayla@gmail.com (T.M.); birincibuse7@gmail.com (B.B.); 3 Department of Biochemistry, Faculty of Veterinary Medicine, Bursa Uludag University, Bursa 16059, Türkiye; abdullahyalcin76@gmail.com (A.Y.); onersonmez@uludag.edu.tr (O.S.); aybikesarioglu0@gmail.com (A.S.-B.); 4 Department of Biochemistry, Health Sciences Institute, Bursa Uludag University, Bursa 16059, Türkiye; elifbbayram@gmail.com; 5 Cellular and Molecular Targeting Laboratory, Faculty of Medicine Experimental Animal Breeding and Research Unit, Bursa Uludag University, Bursa 16059, Türkiye

**Keywords:** capsaicin, cisplatin, combination therapy, drug synergy, non-small-cell lung cancer

## Abstract

**Background/Objectives**: Non-small-cell lung carcinoma (NSCLC) accounts for approximately 85% of lung cancers and remains a leading cause of cancer-related mortality. Although cisplatin is a cornerstone chemotherapeutic agent, its clinical effectiveness is limited by drug resistance and systemic toxicity. Capsaicin (CAP), a bioactive phytochemical derived from chili peppers, has demonstrated anticancer activity in several tumor types and has been investigated as a potential adjunct to conventional chemotherapy. **Methods**: An experimental study was conducted using A549 NSCLC cells and a xenograft mouse model. Cells were treated with CAP (50–300 µM), cisplatin (1 µg/mL), or their combination. Cell proliferation and apoptosis were evaluated using sulforhodamine B (SRB) assay, immunocytochemistry, and Western blot analysis. In vivo, tumor growth inhibition, histopathological alterations, and immunohistochemical expression of Ki-67 and cleaved caspase-3 were assessed. **Results**: Low CAP concentrations (50–100 µM) slightly increased proliferation, whereas concentrations ≥ 150 µM significantly reduced cell viability and induced apoptosis. Cisplatin monotherapy markedly suppressed proliferation and activated apoptosis. Bliss independence analysis demonstrated concentration-dependent synergy between CAP and cisplatin, with maximal synergy scores reaching 28.1 at 100 µM CAP. Combination treatment at CAP concentrations ≥ 150 µM produced the strongest antiproliferative effect in vitro and the highest tumor growth inhibition in vivo (88%). CAP did not further enhance cisplatin-induced apoptosis but significantly reinforced proliferation suppression with reduced Ki-67 expression. **Conclusions**: CAP exhibits biphasic dose-dependent effects in NSCLC and enhances cisplatin antitumor efficacy predominantly through proliferation suppression, supporting its further evaluation as an adjunctive phytochemical in cisplatin-based NSCLC therapy.

## 1. Introduction

Non-small-cell lung carcinoma (NSCLC) is the most prevalent form of lung cancer, accounting for nearly 85% of all cases worldwide [1]. This malignancy is highly heterogeneous, exhibiting diverse molecular features and clinical behaviors that complicate both diagnosis and therapeutic management. Most patients present with advanced-stage disease, contributing to limited treatment options and poor outcomes. The overall five-year survival rate remains approximately 16%, underscoring the critical need for innovative therapeutic strategies [2].

Chemotherapy remains a cornerstone in NSCLC management; however, its clinical utility is limited by systemic toxicities such as nausea, fatigue, and myelosuppression, which compromise treatment adherence and patient quality of life [3].

Cisplatin, one of the most widely used agents, is particularly associated with nephrotoxicity, ototoxicity, and neurotoxicity, highlighting the need for strategies that maintain efficacy while minimizing adverse effects [4,5].

Natural bioactive compounds have emerged as promising adjuncts to conventional chemotherapy, with evidence showing their ability to sensitize cancer cells to treatment and alleviate toxicities [6]. Preclinical studies indicate that combining cisplatin with plant-derived compounds such as polyphenols and flavonoids can potentiate anticancer activity and improve tolerability [7,8]. Such approaches may also address the quality-of-life impairments frequently encountered during cytotoxic therapy.

Among these compounds, capsaicin—the pungent alkaloid derived from chili peppers—has attracted increasing attention for its antineoplastic potential. Initially recognized for its dietary and sensory properties, capsaicin has since been shown to modulate oncogenic signaling and induce cytotoxicity across several cancer types. For example, it triggers apoptosis through the p38 MAPK pathway [9,10], induces autophagy and apoptosis via PI3K/AKT/mTOR inhibition [11,12], and enhances ROS-mediated apoptotic signaling in pancreatic cancer models [13].

Previous studies have also investigated capsaicin in relation to NSCLC and cisplatin-based combination therapy. In NSCLC models, capsaicin was shown to suppress proliferation and induce ferroptosis through regulation of the SLC7A11/GPX4 axis [14]. In addition, recent studies in other epithelial cancer models demonstrated that capsaicin combined with cisplatin can inhibit EMT-associated migration and metastatic behavior through pathways such as Claudin-1/PI3K/AKT/mTOR and EMT/ERK/SERPINB2 signaling [15,16]. Nevertheless, the dose-dependent interaction of capsaicin and cisplatin on proliferation- and apoptosis-related readouts in A549 NSCLC cells, supported by in vivo xenograft validation, remains insufficiently characterized.

Given its natural origin as a bioactive phytochemical, capsaicin remains of interest as a potential adjunct to conventional anticancer therapy. This translational perspective supports further evaluation of capsaicin-containing combination approaches in oncology. Therefore, in the present study, we aimed to investigate the potential of capsaicin in combination with cisplatin in NSCLC, both in vitro and in vivo, to evaluate whether capsaicin could enhance cisplatin-associated antitumor activity in experimental NSCLC models.

## 2. Results

### 2.1. Capsaicin Potentiates the Antiproliferative Effects of Cisplatin in A549 Cells

**Figure 1:**
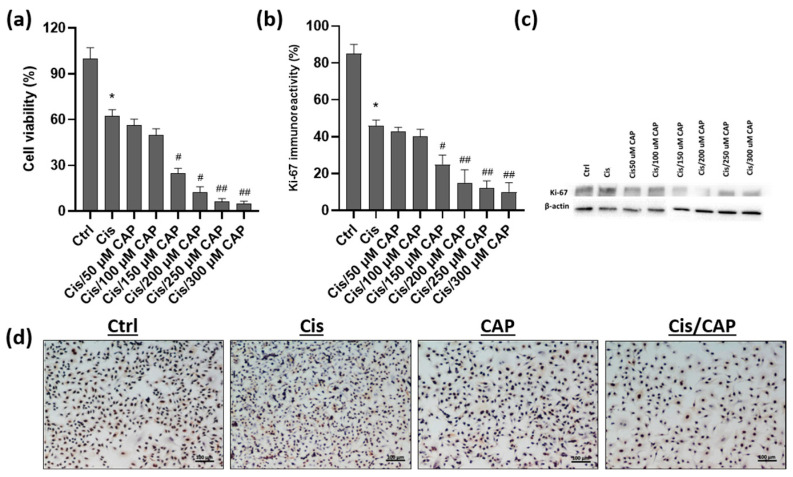
Capsaicin enhances the antiproliferative effects of cisplatin in A549 cells. (**a**) Cell viability was assessed using the SRB assay following treatment with cisplatin (Cis), capsaicin (CAP), or their combinations at the indicated concentrations for 48 h. (**b**) Immunocytochemical quantification of Ki-67 positivity revealed a dose-dependent decrease in proliferative activity with CAP, which was further enhanced when combined with cisplatin. (**c**) Western blot analysis of Ki-67 expression confirmed the immunocytochemical results. β-actin was used as a loading control. Representative blot from three independent experiments. (**d**) Representative immunocytochemical images of Ki-67 expression in control (Ctrl), Cis, 150 µM CAP, and Cis + 150 µM CAP groups. A marked reduction in the proportion of Ki-67- positive nuclei is evident in the combination group compared with monotherapies. The error bars represent the mean  ±  SEM (three replicates). * *p* < 0.05 vs. Ctrl; # *p* < 0.05 vs. Cis; ## *p* < 0.01 vs. Cis.

SRB assay results demonstrated that low doses of CAP (50–100 µM) slightly increased A549 cell viability compared with the control group, whereas a marked reduction in viability was observed at concentrations of ≥150 µM (*p* < 0.05) (Supplementary Figure S1b). Cisplatin treatment alone significantly reduced cell viability relative to control (*p* < 0.05), and the combination with CAP further enhanced this inhibitory effect at doses ≥ 150 µM (Figure 1a). To further assess these findings at the morphological level, light microscopy was performed (Supplementary Figure S1a). Loss of cell–cell junctions and disruption of cellular morphology became evident starting at 200 µM CAP, with more pronounced deterioration at 250–300 µM in combination with cisplatin.

Bliss independence analysis demonstrated a dose-dependent synergistic interaction between capsaicin and cisplatin in A549 cells (Figure 1d). While low-dose capsaicin (50–100 µM) alone induced a modest proliferative response, its combination with cisplatin (1 µg/mL) resulted in a marked reduction in cell viability compared to the expected additive effect. The highest Bliss synergy score was observed at 100 µM capsaicin (+28.1), followed by 150–200 µM concentrations, indicating a favorable combination effect within the tested dose range. At higher capsaicin doses (≥250 µM), synergy scores declined, likely due to a floor effect associated with maximal cytotoxicity.

Ki-67 immunocytochemistry results were consistent with the SRB data. CAP at 50 µM and 100 µM induced a slight, non-significant increase in Ki-67 positivity (*p* > 0.05), while concentrations ≥150 µM led to a dose-dependent reduction (*p* < 0.05). At 250 µM and 300 µM CAP, Ki-67 expression was almost completely abolished (Figure 1b,d and Supplementary Figure S1b). Cisplatin alone significantly decreased Ki-67 levels compared with control (*p* < 0.05), and CAP co-treatment at ≥150 µM further potentiated this effect (Figure 1b,d).

Western blot analysis of Ki-67 expression corroborated these results. Interestingly, CAP at 150 µM alone increased Ki-67 protein expression relative to control, suggesting a paradoxical pro-proliferative effect at this concentration. However, when combined with cisplatin, this increase was suppressed, and Ki-67 levels were markedly reduced across higher CAP concentrations (Figure 1c). Importantly, the 150 µM dose appears to induce a more desirable antiproliferative effect in combination with cisplatin, whereas higher doses (≥200 µM) were associated with profound loss of cell morphology and signs of cytotoxicity, indicating a shift from selective growth inhibition toward non-specific toxicity.

### 2.2. Capsaicin Potentiates Cisplatin-Induced Apoptosis in A549 Cells

**Figure 2:**
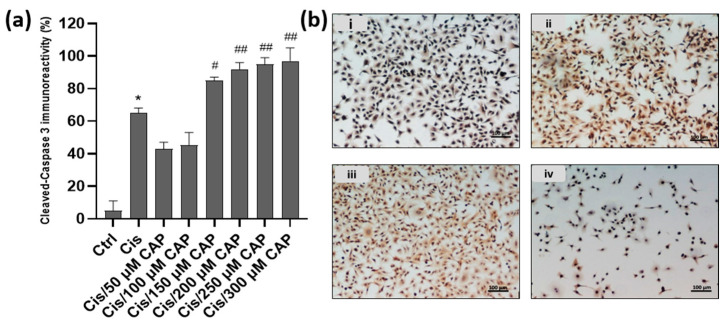
Capsaicin potentiates cisplatin-induced apoptosis in A549 cells. (**a**) Quantitative analysis of cleaved caspase-3 immunoreactivity following treatment with cisplatin (Cis), capsaicin (CAP), or their combinations at the indicated concentrations for 48 h. (**b**) Representative immunocytochemical images of cleaved caspase-3 expression. (**i**) Control (Ctrl), (**ii**) Cis, (**iii**) 150 µM CAP, and (**iv**) Cis + 150 µM CAP. A higher density of cleaved caspase-3-positive cells is evident in the cisplatin and Cis/CAP groups compared with control and CAP alone. The error bars represent the mean  ±  SEM (three replicates). * *p*  <  0.05 vs. Ctrl; # *p*  <  0.05 vs. Cis; ## *p* < 0.01 vs. Cis.

Cleaved caspase-3 expression was evaluated as an indicator of apoptosis (Figure 2a,b). The results demonstrated an inverse trend compared with proliferation markers, thereby reinforcing the overall findings. Low-dose CAP (≤100 µM) did not produce a significant increase in cleaved caspase-3 positivity (Figure 2a) (*p* > 0.05), suggesting a threshold effect. In contrast, CAP at 150 µM and higher concentrations significantly elevated cleaved caspase-3 expression (Figure 2(a,biii)), with a progressive increase at doses ≥ 200 µM (Figure 2(biv)) (*p* < 0.05).

Cisplatin treatment alone markedly increased cleaved caspase-3 expression compared with the control group (Figure 2(a,bii)) (*p* < 0.05). Notably, in the combination groups, a pronounced enhancement of apoptosis became evident at 150 µM CAP (Figure 2(a,biii)), aligning with the Ki-67 findings where synergistic antiproliferative effects also emerged at this concentration. However, at doses above 150 µM, the apoptotic response was accompanied by severe morphological deterioration (Figure 2(biv)), indicating a shift from selective apoptosis toward non-specific cytotoxicity.

### 2.3. Combination of Capsaicin and Cisplatin Significantly Suppresses Tumor Growth in Xenograft Models

**Figure 3:**
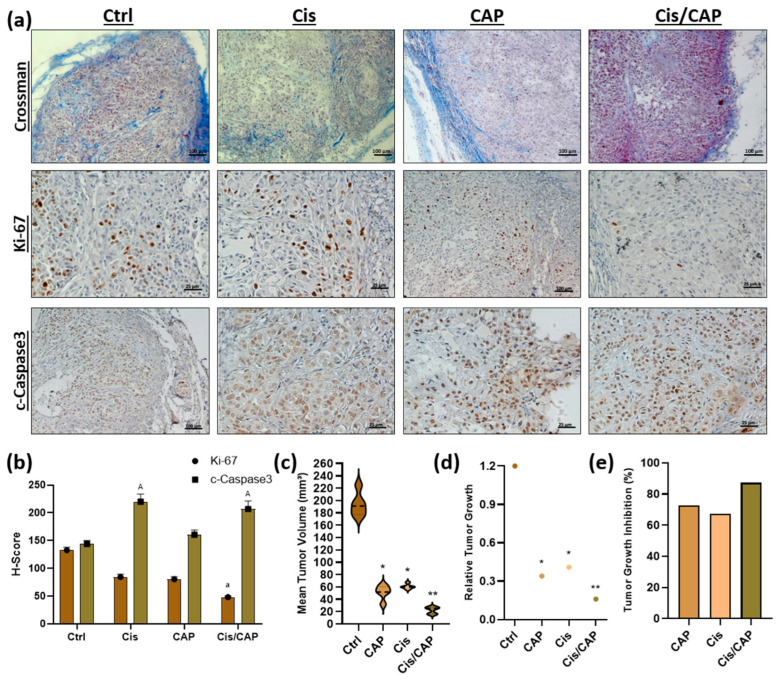
Combination of capsaicin and cisplatin significantly suppresses tumor growth and alters histopathological features in A549 xenograft models. (**a**) Representative histological and immunohistochemical images of xenograft tumors from control (Ctrl), cisplatin (Cis), capsaicin (CAP), and combination (Cis/CAP) groups. Crossman staining demonstrated increased stromal content and collagen deposition, visualized by aniline blue staining, in the Cis and Cis/CAP groups compared with Ctrl and CAP. Ki-67 immunostaining revealed a marked reduction in proliferative activity, particularly in the Cis/CAP group. Cleaved caspase-3 (c-Caspase3) immunostaining showed cytoplasmic and nuclear brown-positive staining, with the most pronounced expression in the Cis and Cis/CAP groups. (**b**) Quantitative H-score analysis of Ki-67 and cleaved caspase-3 immunoreactivity. Cis and Cis/CAP treatments significantly reduced Ki-67 and increased cleaved caspase-3 expression relative to Ctrl. (**c**) Violin plot representation of mean tumor volume in each group, showing a significant reduction in Cis and Cis/CAP groups compared with Ctrl. (**d**) Relative tumor growth values indicate suppressed tumor progression in all treatment groups, with the greatest effect in Cis/CAP. (**e**) Tumor growth inhibition (TGI) percentages for CAP, Cis, and Cis/CAP groups, with the combination producing the highest TGI. The error bars represent the mean ± SEM (five replicates). * *p* < 0.05 vs. Ctrl; ** *p* < 0.01 vs. Ctrl; ^a^ *p* < 0.05 vs. Ctrl for Ki67; ^A^ *p* < 0.05 vs. Ctrl for c-Caspase3.

To evaluate the in vivo relevance of the in vitro findings, a xenograft tumor model was established in immunodeficient mice. Tumor volume, relative tumor growth, tumor growth inhibition (TGI), and histopathological as well as immunohistochemical alterations were assessed following three weeks of treatment (Figure 3b–e). At the end of the treatment period, the mean tumor volume in the control group was 190.23 ± 10.2 mm^3^. CAP monotherapy reduced tumor volume to 51.58 ± 5.6 mm^3^, corresponding to a relative tumor growth value of 0.34 and a TGI of 73% (Figure 3c–e). Similarly, cisplatin monotherapy decreased tumor volume to 62.24 ± 5.0 mm^3^, with a relative tumor growth of 0.41 and a TGI of 68% (Figure 3c–e). Importantly, the combination treatment (Cis/CAP) produced the most pronounced effect, reducing tumor volume to 24.37 ± 2.8 mm^3^, with a relative tumor growth of 0.16 and the highest TGI of 88% (Figure 3c–e). These results demonstrate a statistically significant reduction compared with both the control and cisplatin groups (*p* < 0.05).

Histopathological analysis further corroborated these findings (Figure 3a). In the control and CAP monotherapy groups, tumors appeared as large, cohesive masses with relatively preserved cellular architecture. By contrast, cisplatin and Cis/CAP-treated tumors exhibited increased stromal content and collagen deposition, accompanied by reduced tumor cell density and disrupted tissue integrity. Neoplastic cells in these groups were distributed across multiple smaller foci within the stroma rather than forming a single solid mass (Figure 3a).

Immunohistochemical evaluation provided additional insights. Ki-67 staining confirmed nuclear localization across all groups; however, the Cis/CAP combination displayed a significantly lower percentage of Ki-67-positive tumor cells compared with control (Figure 3a,b, *p* < 0.05). Conversely, cleaved caspase-3 immunoreactivity demonstrated cytoplasmic and nuclear positivity, with the strongest expression observed in both cisplatin and Cis/CAP groups (Figure 3a,b). Quantitative analysis revealed significantly elevated cleaved caspase-3 expression in these groups compared with control and CAP monotherapy (*p* < 0.05), although no significant difference was detected between cisplatin and Cis/CAP (*p* > 0.05, Figure 3b).

Collectively, these in vivo results indicate that CAP enhances the antitumor efficacy of cisplatin through pronounced suppression of tumor proliferation, while apoptotic induction appears to be primarily cisplatin-driven without further augmentation by CAP (Figure 3).

## 3. Discussion

Lung cancer remains a leading cause of cancer-related mortality, and platinum-based chemotherapy continues to represent a central therapeutic modality in NSCLC [2,17]. However, resistance and toxicity limit its long-term efficacy [18]. Thus, novel strategies that combine chemotherapy with bioactive compounds such as capsaicin (CAP) have gained attention for their potential to enhance efficacy and mitigate side effects [18,19]. In the present study, we demonstrate that capsaicin exerts a dose-dependent biphasic effect in A549 NSCLC cells and significantly enhances cisplatin-mediated tumor suppression both in vitro and in vivo.

A549 cells were selected as the experimental model due to their well-characterized molecular background and the extensively documented pharmacological response to cisplatin in this cell line, which provides a reliable platform for evaluating drug–drug interactions. Our findings confirm that low concentrations of CAP (50–100 µM) may exert modest proliferative effects, whereas concentrations ≥ 150 µM induce robust antiproliferative and pro-apoptotic responses [20]. Notably, while maximal Bliss synergy was observed at 100 µM, the most consistent antiproliferative phenotype emerged at 150 µM; therefore, this concentration was considered a biologically informative in vitro threshold concentration for combination treatment. In contrast, CAP concentrations ≥ 150 µM significantly suppressed proliferation and triggered apoptotic signaling, consistent with previous reports demonstrating p53 activation and caspase-dependent pathways as well as modulation of cellular redox balance and deacetylase activity in lung cancer models [21,22]. This biphasic pattern underscores the importance of dose optimization, as subtherapeutic exposure may activate survival pathways, while higher concentrations effectively suppress tumor growth. Importantly, Bliss independence analysis revealed a concentration-dependent synergistic interaction between CAP and cisplatin, with maximal synergy observed at intermediate CAP doses. These data suggest that CAP may act as an adjunctive component in CAP/cisplatin combination treatment rather than merely exerting additive cytotoxicity. The low-dose proliferative tendency of CAP highlights the importance of careful dose optimization. Although this effect may reflect a hormetic or adaptive cellular response, the underlying mechanism was not directly examined in the present study. Therefore, future studies should clarify the molecular basis and safety implications of low-dose CAP exposure. Accordingly, 150 µM CAP should not be considered a definitively optimal concentration, but rather a biologically informative in vitro threshold concentration in our experimental setting. Although this dose marked the onset of consistent antiproliferative activity, its physiological relevance and safety window remain unclear. Therefore, the present findings should be interpreted as experimental evidence of CAP-supported cisplatin activity rather than direct evidence for clinical applicability, and future studies should include pharmacokinetic evaluation, normal cell models, and comprehensive systemic toxicity analyses.

Consistent with its well-established DNA-damaging mechanism, cisplatin monotherapy induced marked antiproliferative and pro-apoptotic responses both in vitro and in vivo [23]. Co-treatment with CAP further enhanced cisplatin-mediated growth suppression, with the most pronounced effects observed at intermediate CAP concentrations. Although CAP did not significantly augment cisplatin-induced caspase-3 activation in vivo, the substantial reduction in Ki-67 expression indicates that its primary contribution lies in reinforcing proliferation blockade rather than amplifying apoptotic signaling. These findings suggest a complementary mode of action in which CAP modulates cell-cycle dynamics to potentiate platinum efficacy. Based on our experimental readouts, the enhanced antitumor effect of the Cis/CAP combination appears to be mainly associated with reinforced proliferation blockade rather than a further increase in apoptosis. This interpretation is supported by the marked reduction in SRB viability and Ki-67 expression, together with the highest tumor growth inhibition observed in vivo. In contrast, cleaved caspase-3 activation was prominent in both cisplatin and Cis/CAP groups, but was not further increased by the combination in vivo. Therefore, capsaicin may enhance cisplatin efficacy by limiting the proliferative capacity of tumor cells and thereby shifting the treatment response toward growth suppression and cisplatin-driven apoptosis. Since upstream signaling molecules were not directly examined in this study, this explanation should be regarded as a data-supported biological interpretation rather than a confirmed molecular pathway.

An important limitation of the present study is that the effects of CAP and the CAP/cisplatin combination on non-cancerous cells and healthy tissues were not directly evaluated. Although no treatment-related mortality or animal exclusion was observed in vivo, detailed systemic toxicity analyses, such as serum biochemical testing and histopathological examination of major organs, were not performed. Therefore, future studies should assess the selectivity and safety profile of CAP-containing combinations using normal cell models and comprehensive in vivo toxicity evaluations.

Beyond cisplatin, capsaicin has demonstrated synergy with other chemotherapeutic agents in preclinical studies. Notably, a recent investigation in triple-negative breast cancer models revealed that capsaicin, when combined with doxorubicin, more effectively reduced primary tumor burden and metastasis, while improving survival outcomes—potentially via modulation of TGF-β signaling [24]. Moreover, a comprehensive review of gynecological cancers highlighted capsaicin’s role in enhancing chemosensitivity in cervical and ovarian cancer models, suggesting its broad potential in improving therapeutic responses [25]. Together with our findings, these data support the potential of capsaicin as an adjunctive component in combination-based anticancer strategies. Recent studies have further supported the potential of capsaicin as an adjunctive agent in combination-based cancer therapy, including cisplatin-containing treatment settings [26,27].

Mechanistically, CAP has been reported to modulate multiple oncogenic pathways, including p53 activation, MAPK signaling, and PI3K/AKT/mTOR suppression, which are central to tumor survival and chemoresistance [10,19,24]. In cisplatin-resistant models, CAP has been shown to restore drug sensitivity through degradation of resistance-associated proteins and inhibition of cytoprotective autophagy [28,29]. Although previous studies have linked capsaicin to p53, MAPK, and PI3K/AKT/mTOR-related pathways, these signaling axes were not directly examined in the present study. Therefore, our interpretation is limited to the experimental readouts assessed here, including SRB viability, Ki-67 expression, cleaved caspase-3 immunoreactivity, and tumor growth inhibition, and should not be considered a confirmed molecular mechanism.

Beyond its direct anticancer effects, capsaicin has also been investigated for its potential to modulate chemotherapy-associated toxicities [30,31]. Although such supportive effects were not evaluated in the present study, these observations further underscore the translational interest in CAP as an adjunct to conventional chemotherapy. Although 150 µM CAP was identified as the most biologically informative in vitro threshold for combination treatment, this concentration is relatively high and should not be directly extrapolated to clinical exposure. Potential off-target effects on non-malignant cells, including cellular stress or non-specific cytotoxicity, cannot be excluded. Therefore, future studies using normal lung epithelial cells and comprehensive toxicity assays are required to define the selectivity and safety window of CAP/cisplatin treatment.

Histopathological evaluation of xenograft tumors revealed distinct architectural differences among treatment groups. While control and CAP monotherapy groups formed compact tumor masses, cisplatin- and combination-treated tumors exhibited increased stromal deposition and reduced tumor cell density. Although multifocal distribution was observed in treated groups, this may partially reflect variability in treatment timing and tumor excision. However, stromal content and collagen deposition were evaluated qualitatively by Crossman staining, and no quantitative morphometric analysis of collagen fiber area was performed. Therefore, it cannot be concluded whether CAP further increased collagen deposition compared with cisplatin alone. The increased stromal content observed in cisplatin-containing groups should be interpreted cautiously, as it may reflect therapy-associated stromal alteration or replacement fibrosis-like change rather than a uniformly favorable adaptive remodeling response. Future studies using quantitative collagen morphometry and additional stromal markers are required to clarify the contribution of CAP to tumor stromal changes.

Consistent with these findings, combination therapy achieved the highest tumor growth inhibition (88%) in vivo, together with marked suppression of proliferative activity. Collectively, these findings support the potential of CAP as an adjunct to cisplatin-based treatment in this xenograft model, mainly by reinforcing antiproliferative effects.

## 4. Materials and Methods

### 4.1. Cell Culture and Drug Treatment

The A549 human non-small-cell lung cancer (NSCLC) cell line was obtained from the American Type Culture Collection (ATCC®, CRM-CCL-185™, Manassas, VA, USA), and experimental procedures were conducted beginning with passage 12 to ensure cell line stability. The human lung carcinoma cell line was cultured in RPMI 1640 medium (ATCC, 30-2001, Manassas, VA, USA) supplemented with 10% (*v*/*v*) fetal bovine serum (FBS; Gibco, Thermo Fisher Scientific, Waltham, MA, USA) and 1× penicillin-streptomycin (100 IU/mL, 100 µg/mL; Gibco, Thermo Fisher Scientific, Waltham, MA, USA). Cells were maintained at 37 °C in a humidified incubator with 5% CO_2_ and 95% air atmosphere. To investigate the combinatorial effects of capsaicin (CAP) and cisplatin (Cis), A549 cells were subjected to four treatment conditions. In the vehicle control group, cells were treated with <0.2% (*v*/*v*) dimethyl sulfoxide (DMSO; Sigma-Aldrich, D4540, St. Louis, MO, USA), corresponding to the solvent used for CAP. In the Cis group, cells were exposed to 1 µg/mL cisplatin (Sigma-Aldrich, C2210000, St. Louis, MO, USA) dissolved in molecular-grade water. In the CAP group, cells received CAP (Sigma-Aldrich, 404-86-4, St. Louis, MO, USA) at final concentrations of 50, 100, 150, 200, 250, or 300 µM, prepared in DMSO with a final concentration not exceeding 0.2% (*v*/*v*). In the combination group (Cis/CAP), 1 µg/mL cisplatin was administered together with each concentration of CAP. All treatments were carried out under standard culture conditions for 48 h. The capsaicin concentrations used in this study (50–300 µM) were selected as a wide-range, stepwise incremental dose panel designed to capture the compound’s biphasic behavior previously described in NSCLC models. This approach allowed precise identification of low-dose proliferative effects as well as the transition to growth-inhibitory and cytotoxic thresholds. Cisplatin was administered at 1 µg/mL, a commonly used reference dose [32] that produces measurable but non-lethal cytotoxicity in A549 cells, enabling reliable assessment of combinational effects. We used only the A549 cell line because it represents the most widely used and genetically well-defined model of NSCLC for cisplatin-based pharmacology. Restricting the study to a single line reduced biological variability and enabled detailed mechanistic analysis, which forms the basis for future multi-line validation studies.

### 4.2. Sulforhodamine B (SRB) Assay

Cell viability was assessed using the sulforhodamine B (SRB) assay, which quantifies total cellular protein content as a proxy for cell density. A549 cells were seeded into 96-well plates at a density of 5000 cells per well and allowed to adhere overnight. Cells were then treated according to the experimental groups described above and incubated for 48 h. Following treatment, cells were fixed by the addition of cold 10% trichloroacetic acid (TCA; Thermo Fisher Scientific, J62355.AP, Waltham, MA, USA) and incubated at 4 °C for 1 h. The plates were then rinsed thoroughly with tap water and allowed to air-dry. Fixed cells were stained with 0.4% (*w*/*v*) SRB solution (Thermo Fisher Scientific, S1307, Waltham, MA, USA) for 60 min at room temperature. Unbound dye was removed by washing with 1% (*v*/*v*) acetic acid, and the plates were air-dried again. The protein-bound dye was solubilized using 10 mM Tris base (pH ~10.5; Merck, 77-86-1, Darmstadt, Germany), and absorbance was measured at 510 nm using an Epoch™ Microplate Spectrophotometer (BioTek Instruments, Winooski, VT, USA).

Drug–drug interactions were evaluated using the Bliss independence model based on % cell viability values. Expected combination effects were calculated as EBliss = EA × EB, and synergy scores were defined as the difference between observed and expected inhibition. Positive scores indicate synergistic interactions.

### 4.3. Immunocytochemistry (ICC)

A549 cells were seeded onto sterile glass coverslips placed in 24-well culture plates at an appropriate density and allowed to adhere overnight. Cells were then treated according to the experimental groups described in the drug treatment section. After 48 h of incubation, the cells were washed three times with phosphate-buffered saline (PBS; Thermo Fisher Scientific, 10010023, Waltham, MA, USA) and fixed with 4% paraformaldehyde (Thermo Fisher Scientific, J62478.AK, Waltham, MA, USA) for 15 min at room temperature. Permeabilization was performed using 0.1% Triton X-100 (Thermo Fisher Scientific, HFH10, Waltham, MA, USA) for 10 min, followed by blocking with a commercial blocking reagent (Vector Laboratories, MP7401, Newark, CA, USA) for 20 min to minimize nonspecific antibody binding. Cells were then incubated overnight at 4 °C with primary antibodies diluted in antibody diluent (Thermo Fisher Scientific, 003118, Waltham, MA, USA): anti-Ki-67 (to assess cell proliferation; Abcam, ab92742, Cambridge, UK) and anti-cleaved caspase-3 (to evaluate apoptosis; Cell Signaling Technology, 9661, Danvers, MA, USA). The next day, cells were incubated with an appropriate biotinylated secondary antibody (Vector Laboratories, MP7401, Newark, CA, USA) for 30 min at room temperature. Immunoreactivity was visualized using DAB chromogen (Abcam, ab64238, Cambridge, UK) for 5 min, followed by counterstaining with Harris hematoxylin (Sigma-Aldrich, HHS32, St. Louis, MO, USA) for 2 min. Coverslips were mounted onto glass slides, and immunostaining was visualized and imaged using a Nikon Eclipse 80i microscope (Nikon Corporation, Tokyo, Japan) equipped with plan achromat objectives. For semi-quantitative analysis, five non-overlapping fields were randomly selected from each sample, and the percentage of positively stained cells was independently evaluated by two blinded investigators.

### 4.4. Western Blot Analysis

Expression levels of specific proteins in total cell lysates were evaluated by Western blotting. Briefly, cells were first washed with ice-cold phosphate-buffered saline (PBS) and lysed in a buffer containing 50 mM Tris-HCl (pH 7.5; Thermo Fisher Scientific, 15567027, Waltham, MA, USA), 150 mM NaCl (Thermo Fisher Scientific, AM9760G, Waltham, MA, USA), 1 mM EDTA (Thermo Fisher Scientific, AM9260G, Waltham, MA, USA), 5 mM MgCl_2_ (Thermo Fisher Scientific, AB0359, Waltham, MA, USA), 0.5% NP-40 (Thermo Fisher Scientific, 85124, Waltham, MA, USA), 0.5% Triton X-100 (Thermo Fisher Scientific, Waltham, MA, USA), and protease/phosphatase inhibitor cocktails. The lysates were centrifuged at 10,000× *g* for 5 min at 4 °C to remove insoluble debris, and the supernatants were collected. Protein concentrations were determined using the bicinchoninic acid (BCA) assay (Pierce™ BCA Protein Assay Kit, Thermo Fisher Scientific, 23225, Waltham, MA, USA) by measuring absorbance at 562 nm.

Equal amounts of protein (25 µg) from each sample were mixed with SDS-PAGE sample buffer (1:1) (Thermo Fisher Scientific, LC2676, Waltham, MA, USA), denatured at 95 °C for 5 min, and separated on 12% SDS-polyacrylamide gels. Proteins were subsequently transferred onto polyvinylidene difluoride (PVDF) membranes (Thermo Fisher Scientific, STM2006, Waltham, MA, USA). Membranes were blocked for 1 h at room temperature with 5% non-fat dry milk in TBS-T (Tris-buffered saline with 0.1% Tween-20), followed by overnight incubation at 4 °C with a primary antibody against Ki-67 (Abcam, ab92742, Cambridge, UK). After washing with TBS-T, membranes were incubated for 1 h at room temperature with horseradish peroxidase (HRP)-conjugated goat anti-rabbit secondary antibody. Protein bands were visualized using enhanced chemiluminescence substrate (ECL Prime, GE Healthcare, RPN2232, Chicago, IL, USA) and detected with the ChemiDoc MP Imaging System (Bio-Rad Laboratories, Hercules, CA, USA). β-actin (Cell Signaling Technology, 3700, Danvers, MA, USA) was used as a loading control, and bands were analyzed using Image Lab™ software (Bio-Rad Laboratories, Hercules, CA, USA). All washing steps between antibody incubations were performed with TBS-T.

### 4.5. Xenograft Experiments in Mice

A total of 20 male immunodeficient CD-1 Nude mice (4–6 weeks old), with identical genetic backgrounds, were purchased from Kobay DHL A.S. (Ankara, Turkey). All animal care and experimental procedures were performed at the Uludag University Center for Laboratory Animal Research and Production (DENHAB) under approval of the Local Ethics Committee for Animal Experiments (HADYEK; protocol no: 2020-04/02). To establish subcutaneous xenograft tumors, A549 cells (2 × 10^6^) were suspended in a 1:1 ratio with Matrigel (Corning, 356237, Corning, NY, USA) and injected into the right flank of each mouse in a total volume of 0.2 mL. Once tumors reached an average volume of 150 mm^3^, mice were randomly allocated into four groups (Control, CAP, Cisplatin, and Combination; *n* = 5 per group) using random number assignment to ensure unbiased distribution. Investigators responsible for tumor measurement and histopathological assessment were blinded to group allocation during data collection and analysis in order to minimize bias. The vehicle control group received corn oil, used as the capsaicin solvent, diluted in 0.1 mL phosphate-buffered saline (PBS) via intraperitoneal injection three times per week. The CAP group was treated with 5 mg/kg capsaicin (CAP) dissolved in corn oil and administered intraperitoneally three times weekly [33]. The cisplatin (Cis) group received 3 mg/kg cisplatin via intraperitoneal injection once weekly [11]. For in vivo experiments, capsaicin (5 mg/kg) and cisplatin (3 mg/kg) doses were chosen based on published tolerability profiles and prior efficacy data. In the combination group (Cis + CAP), animals received both 3 mg/kg cisplatin (once per week) and 5 mg/kg CAP (three times per week) through intraperitoneal administration. Mice were monitored regularly for tumor growth, general health status, and body weight throughout the course of the treatment.

Throughout the treatment period, mice were weighed twice weekly to monitor changes in body weight, and tumor dimensions (length and width) were measured at the same frequency using calipers. Tumor volume (V) was calculated using the standard formula: V = (length × width^2^)/2. Additionally, relative tumor growth was determined by dividing the tumor volume on the final day of the experiment (day *n*) by the volume on day 0 (V*_n_*/V_0_), and tumor growth inhibition (TGI) was calculated as follows: TGI (%) = [1 − (ΔT/ΔC)] × 100, where ΔT and ΔC represent the mean tumor volume increase in the treatment and control groups, respectively. At the end of the three-week treatment period, mice were euthanized under general anesthesia with an intraperitoneal injection of a xylazine/ketamine combination at 0.1 mL/100 g, followed by cervical dislocation. Tumors were carefully excised, weighed, and recorded for final tumor burden assessment.

### 4.6. Histopathological and Immunohistochemical Analysis

Excised tumor tissues were fixed in 10% neutral buffered formalin and subsequently processed using routine histological techniques. Following dehydration and clearing, samples were embedded in paraffin, and 5 µm-thick sections were obtained. For general morphological evaluation, sections were stained with Crossman’s modified trichrome and examined under a Nikon Eclipse 80i light microscope (Nikon Corporation, Tokyo, Japan), with representative images captured.

Immunohistochemical (IHC) analyses were performed on serial sections to evaluate cellular proliferation and apoptosis. After deparaffinization and rehydration, antigen retrieval was performed using microwave heating. Endogenous peroxidase activity was quenched, and nonspecific binding was blocked using ImmPRESS® HRP Anti-Rabbit IgG (Vector Laboratories, MP7401, Newark, CA, USA) for 20 min. For detection of proliferative activity, sections were incubated overnight at 4 °C with Ki-67 primary antibody (Abcam, ab92742, Cambridge, UK), followed by incubation with the appropriate secondary antibody according to the manufacturer’s instructions. Apoptosis was assessed using a cleaved caspase-3 primary antibody (Cell Signaling Technology, 9661, Danvers, MA, USA) under identical conditions. In both analyses, immunoreactivity was visualized using a DAB chromogen (Abcam, ab64238, Cambridge, UK), and counterstaining was performed with Harris hematoxylin (Sigma-Aldrich, HHS32, St. Louis, MO, USA). Stained slides were examined under light microscopy, and representative fields were documented.

### 4.7. Statistical Analysis

For immunocytochemical analyses, each antibody staining was evaluated by counting 100 cells in five randomly selected fields per slide. The percentage of positively stained cells was calculated based on the ratio of immunoreactive cells to the total number of counted cells. All in vitro experiments were performed in three independent biological replicates.

For immunohistochemical evaluation of tumor sections, staining intensity was assessed using the H-score method. In each slide, five distinct microscopic fields were analyzed, and cells were scored based on staining intensity in four categories: no staining (0), weak (+, score 1), moderate (++, score 2), and strong (+++, score 3). The H-score was calculated using the formula: H-score = ∑(I × PC), where I represents the staining intensity and PC the percentage of cells exhibiting that intensity. All histological assessments were performed simultaneously by two independent blinded observers to minimize bias.

All statistical analyses were conducted using IBM SPSS Statistics software (version 23, IBM Corp., Armonk, NY, USA) with a 95% confidence level. After testing for homogeneity, data were found not to follow a normal distribution. Therefore, non-parametric tests were applied: the Kruskal–Wallis test was used for overall group comparisons, and pairwise differences were analyzed using the Mann–Whitney U test. Data are presented as mean ± standard error of the mean (SEM), and a *p*-value ≤ 0.05 was considered statistically significant.

## 5. Conclusions

Our findings demonstrate that capsaicin exhibits dose-dependent effects in NSCLC models, with low concentrations showing a slight proliferation-enhancing tendency and higher concentrations producing marked growth suppression. In combination with cisplatin, CAP enhanced antitumor activity mainly by reinforcing antiproliferative effects, as supported by reduced SRB viability, decreased Ki-67 expression, and pronounced tumor growth inhibition in vivo. However, apoptosis appeared to be predominantly cisplatin-driven, since cleaved caspase-3 activation was not further increased by the combination compared with cisplatin alone. Therefore, CAP should be considered a potential adjunct to cisplatin-based treatment in this experimental setting rather than a definitively established chemosensitizing agent. Further studies including normal cell models, pharmacokinetic evaluation, systemic toxicity assessment, and pathway-specific mechanistic analyses are required to clarify its safety, selectivity, and translational relevance.

## Data Availability

The data presented in this study are contained within the article and Supplementary Materials.

## Supplementary Materials

The following supporting information can be downloaded at: https://www.mdpi.com/article/10.3390/ph19060884/s1, Figure S1: Dose-dependent effects of capsaicin on A549 cell morphology, viability, proliferation, and apoptosis.

## Abbreviations

BCA	Bicinchoninic acid
CAP	Capsaicin
Cis	Cisplatin
DAB	3,3′-Diaminobenzidine
DMSO	Dimethyl sulfoxide
ECL	Enhanced chemiluminescence
EDTA	Ethylenediaminetetraacetic acid
FBS	Fetal bovine serum
HRP	Horseradish peroxidase
ICC	Immunocytochemistry
IHC	Immunohistochemistry
NSCLC	Non-small-cell lung cancer
PBS	Phosphate-buffered saline
PVDF	Polyvinylidene difluoride
ROS	Reactive oxygen species
SEM	Standard error of the mean
SRB	Sulforhodamine B
TBS-T	Tris-buffered saline with Tween-20
TCA	Trichloroacetic acid
TGI	Tumor growth inhibition
