# Supplementary figures and images for: Capsaicin as a Phytochemical Adjuvant Enhancing Cisplatin Efficacy in Nsclc: In Vitro and In Vivo Evidence
Source: Pharmaceuticals (Basel). 2026 Jun 1;19(6):884. doi: 10.3390/ph19060884 (PMC13306043; doi:10.3390/ph19060884)

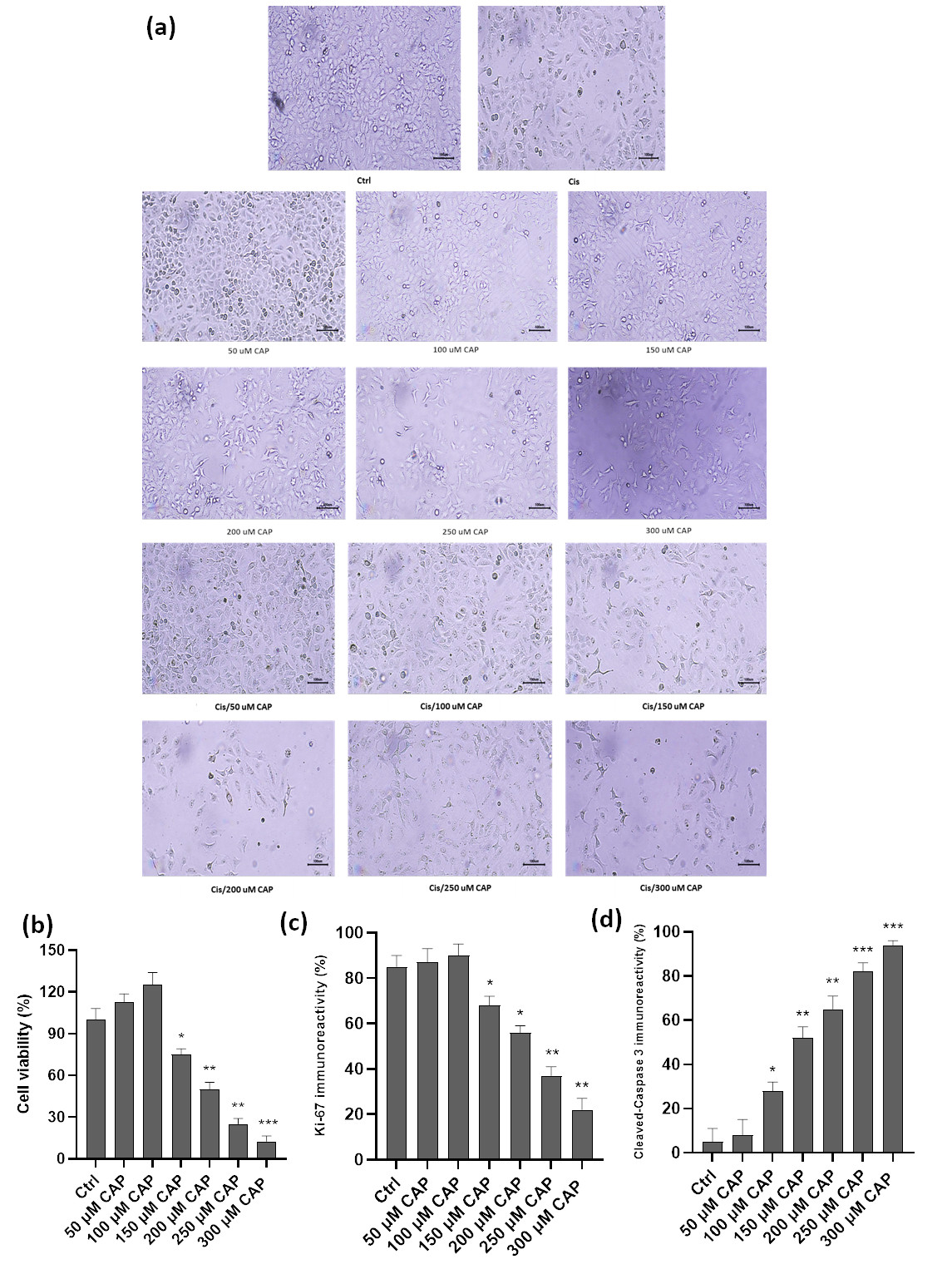

Supplement: Supplementary file 1 [file pharmaceuticals-19-00884-s001.zip › pharmaceuticals-4274967-supplementary.jpg]
